# Lipid Self-Assemblies under the Atomic Force Microscope †

**DOI:** 10.3390/ijms221810085

**Published:** 2021-09-18

**Authors:** Aritz B. García-Arribas, Félix M. Goñi, Alicia Alonso

**Affiliations:** Instituto Biofisika (CSIC, UPV/EHU), Universidad del País Vasco, 48940 Leioa, Spain; aritzgarciaar@hotmail.com (A.B.G.-A.); felix.goni@ehu.es (F.M.G.)

**Keywords:** lipid assemblies, cell membranes, model membranes, nanodomains, atomic force microscopy, supported planar bilayers, phospholipids, sphingolipids

## Abstract

Lipid model membranes are important tools in the study of biophysical processes such as lipid self-assembly and lipid–lipid interactions in cell membranes. The use of model systems to adequate and modulate complexity helps in the understanding of many events that occur in cellular membranes, that exhibit a wide variety of components, including lipids of different subfamilies (e.g., phospholipids, sphingolipids, sterols…), in addition to proteins and sugars. The capacity of lipids to segregate by themselves into different phases at the nanoscale (nanodomains) is an intriguing feature that is yet to be fully characterized in vivo due to the proposed transient nature of these domains in living systems. Model lipid membranes, instead, have the advantage of (usually) greater phase stability, together with the possibility of fully controlling the system lipid composition. Atomic force microscopy (AFM) is a powerful tool to detect the presence of meso- and nanodomains in a lipid membrane. It also allows the direct quantification of nanomechanical resistance in each phase present. In this review, we explore the main kinds of lipid assemblies used as model membranes and describe AFM experiments on model membranes. In addition, we discuss how these assemblies have extended our knowledge of membrane biophysics over the last two decades, particularly in issues related to the variability of different model membranes and the impact of supports/cytoskeleton on lipid behavior, such as segregated domain size or bilayer leaflet uncoupling.

## 1. Introduction: Membranes

The cell membrane concept has evolved along the decades, from the first ideas on how cells need to isolate themselves from the surrounding medium [1], in the late 19th century, to the groundbreaking proposal of the fluid-mosaic model [2] in 1972. Furthermore, over the last 50 years, new findings have modified this model towards a greater degree of complexity [3,4,5,6], in which aspects such as protein–protein interactions and lipid leaflet asymmetry have gained importance [7]. Among these relevant novelties, bilayer heterogeneity is perhaps one of the most intriguing, as lipids may be present in different phases and govern the properties of the membrane at different local points [8]. The lipid raft hypothesis has been one of the most impactful contributions to this area [9], leading to the current concept of nanodomains. The somehow related concept of lipid phase refers to the physical state of lipid assemblies in aqueous media, in the same way as H_2_O may be present in either solid, liquid or gas phases, or sometimes in a combination of them (e.g., an ice cube floating in water). However, lipids are often found in phases whose properties are intermediate between solid and liquid, the so-called mesophases.

**Figure 1:**
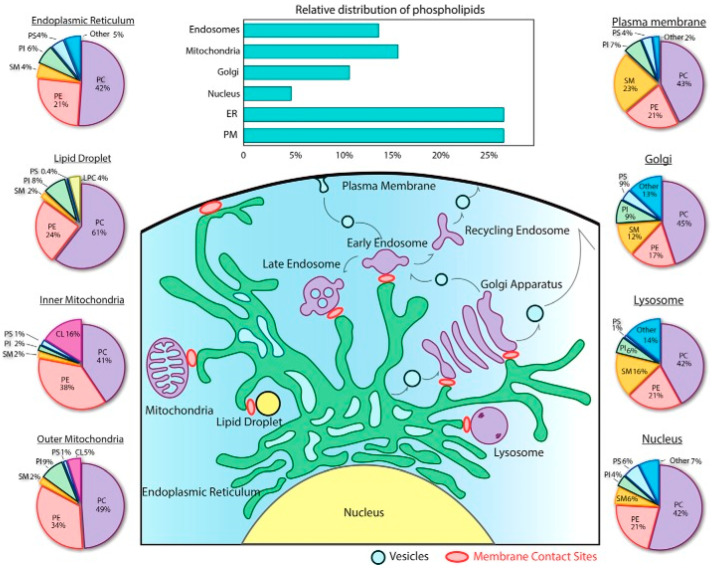
Glycerophospholipid composition of organelles. The bar graph highlights the subcellular distribution of glycerophospholipids between the different organelles in baby hamster kidney cells. The pie charts display the relative abundance of each lipid class in organelles, based on composite data from rat hepatocytes and (for lipid droplets) from murine hepatocytes. The figure shows the variability of lipids, with phosphatidylcholines (PCs) and phosphatidylethanolamines (PEs) as the most predominant species. Adapted from Yang, et al. [14].

The most common lipid phases in biology are the lamellar ones (bilayers), particularly the liquid-disordered (‘fluid’, or ‘liquid-crystalline’, the most prevalent one) and the liquid-ordered ones, the latter usually related to the presence of cholesterol [10]. Bilayers often exhibit a melting temperature (T_m_), below which an additional, solid-ordered lamellar phase (commonly known as ‘gel phase’) exists [11]. T_m_ depends on the lipids present in the system. This is relevant as lipid distribution is extremely variable in membranes, even more if we compare not only different cell lines but also different organelles within a single cell (Figure 1), or distinct lipid leaflets of the very same membrane (membrane lipid asymmetry). In addition other, non-lamellar, lipid morphologies—e.g., hexagonal, or cubic [3]—may be present transiently (e.g., in membrane fusion and fission processes, or to facilitate protein insertion in membranes [12,13]), which greatly increase membrane complexity and variability.

## 2. Lipids: Building Blocks and More

**Figure 2:**
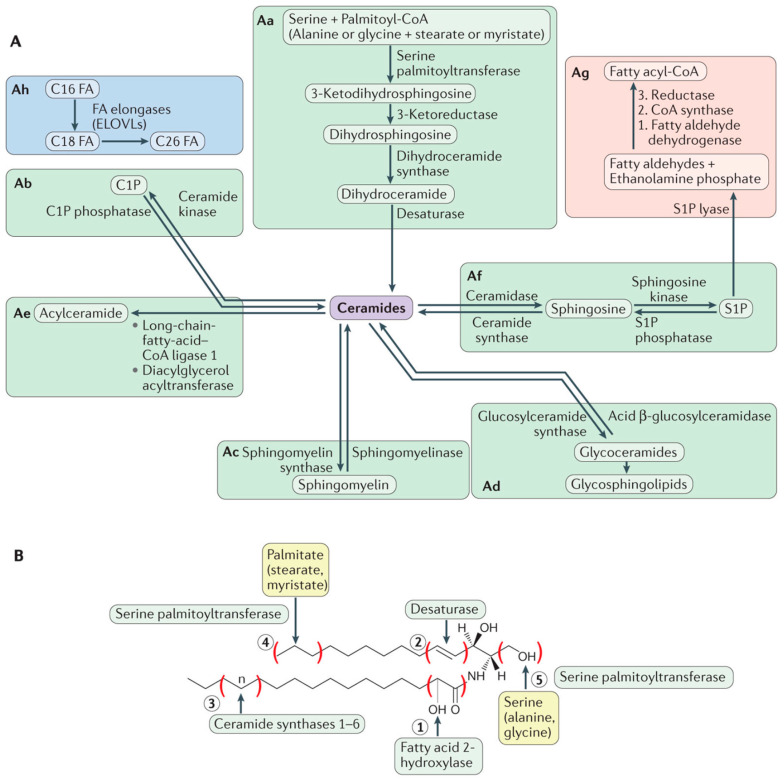
Overview of sphingolipid metabolism. Being the precursors of all complex sphingolipids, ceramides constitute a family of closely related molecules that contain a sphingoid base and an amino-linked fatty acid. Ceramide can be generated through different pathways but most of them can also operate in reverse and use ceramide to produce other metabolic products (**A**). In addition, different enzymes may introduce variations to the basic structure, increasing variability (**B**). From Hannun and Obeid [28].

Lipids, particularly phospholipids and sterols, play a fundamental role as the building blocks of the cell membrane lipid matrix. This occurs because of the amphiphilic nature of these molecules, that favors their self-assembly in aqueous media [2,3]. Lipid self-assembly, mainly driven by entropy, is the basis for the spontaneous formation of not only cell membranes but also of the lipid bilayers used as ‘model membranes’ in biophysical studies (see below). Moreover, a widespread misconception of lipids in membranes is that they have only a strictly structural function. As stated previously, the structural concept of cell membranes has evolved over the last decades towards a more complex perspective. Accordingly, the role of lipids has also been expanded with the discovery of their bioactive effects, particularly for sphingolipids, a subfamily of lipids structurally based on a sphingosine backbone that undergo many metabolic modifications (Figure 2). While sphingolipids had been discovered over a century ago [15], they were considered as simply structural lipids, thus attracting scarce interest for 100 years. However, their bioactive role was described in the 80s of the past century [16,17] suddenly bringing them to the spotlight as bioactive protagonists. Sphingolipids such as ceramides [18] have a pro-apoptotic role [19,20] and are used as chemotherapeutic agents for cancer treatments [21,22]. Other sphingolipids, like ceramide-1-phosphate, have the opposite tendency, as they constitute pro-survival signals [23] and may be of interest as pro-inflammatory agents [24]. Moreover, lipids have more recently attracted a renewed interest as the metabolic impact of lipid droplets (lipid reservoirs within cells) has been unveiled [25]. Lipids could also play a role in the immune response (e.g., for virus-infected cells [26]) and some reports point to a functional relationship between lipid droplets and sphingolipid metabolism [27].

Although the biological impact of lipid signaling is currently beyond discussion, how does this relate to cell membrane structure? That is perhaps the most important question regarding this topic, not yet fully understood. What we actually do know is that sphingolipids have (usually) a higher T_m_ than their glycerolipid counterparts, and accordingly the former have a tendency to form membrane domains. This makes them extremely impactful for the biophysical properties of membranes [18]. Ceramides, due to their high hydrophobicity as well as their high T_m_ values, tend to form gel phase platforms [29,30,31,32] depending on their carbon chain length [33,34,35], while also inducing membrane permeability [36,37,38,39,40] and lipid flip-flop (loss of bilayer asymmetry) [41]. Sphingomyelins (which are non-bioactive *per se*) are considered to be an integral part of the liquid-ordered domains as they have a strong affinity for cholesterol [42], but they are also reservoirs for quick ceramide generation via sphingomyelinases [43]. This means that ceramide formation may not only cause ceramide-enriched ‘gel’ domains but also contribute to ‘liquid-ordered’ nanodomains to coalesce due to sphingomyelin cleavage and recruitment. In fact, a competition between cholesterol and ceramide for sphingomyelin binding was proposed as they shared the tendency to occupy the same hydrophobic pockets for sphingomyelin interaction [44,45,46], but later reports indicated that this displacement might not be occurring in many relevant cases where both molecules can be accommodated and interact in a stabilizing manner [30,47,48,49], as discussed in a previous report [50].

To further assess the relationship between membrane structure and the bioactive (apoptotic) effects of its constituting lipids, the current consensus hypothesis is that sphingolipids play a key role in the formation of the transient MOMP (Mitochondrial Outer Membrane Pore), essential for cytochrome c translocation from mitochondria to cytosol, thereby triggering the intrinsic apoptotic cascade [51]. For this purpose, ceramides have been proposed to interact with Bcl-2 family apoptotic proteins such as Bak [52] and Bax [53]. How ceramides are transported to the mitochondrial outer membrane from the endoplasmic reticulum is an important target of current research [54]. In addition, ceramide platforms have also been proposed to interact with Fas [55] for receptor-mediated extrinsic apoptosis, which is non-cytochrome c dependent.

## 3. Membrane Biophysics: Model Membranes

The study of membrane structural properties and characterization is usually named ‘membrane biophysics’. The complexity of membranes in living cells requires the use of model membranes to understand the basic principles of molecular interactions in those structures. ‘Model membranes’ is a wide concept that may include a vast array of simplified systems, from those composed of a single lipid, to lipid membrane extracts that may also include proteins. One common feature of model membranes is that they are dependent on the self-assembly properties of membrane lipids, mainly phospholipids and cholesterol. The usual approaches in membrane biophysics through model membranes are: (i) to gradually increase complexity of the systems when the most basic ones have been understood (a Cartesian approach) and/or (ii) to compare different sets of systems where only one component (or parameter) has been changed (a comparative approach).

Lipid polymorphism is a factor that increases even more the possibilities for model membranes. As previously stated, lipids have their own phase behavior, and lipid geometry is very important in this respect, as it governs membrane curvature, thus the mode(s) of lipid self-assembly [56]. Depending on the intrinsic curvature of lipids present in a sample, different non-lamellar structures can be induced, such as micelles, tubules (tethers), or hexagonal (normal or inverted) phases [57,58].

The most commonly used model system in membrane biophysics is the liposome, as it shares the basic structural principles of a biological membrane: a lamellar lipid structure which isolates an internal aqueous medium from an external one. While the liposome does not present the internal architecture of a cell (it lacks cytoskeleton, for instance) and usually forms spherical structures, it allows a controlled lipid environment as we define the lipid composition of the system. Liposomes are also used as drug carriers in pharmaceutical industry [59,60]—e.g., in cancer treatments [61] or COVID-19 vaccines, from Pfizer (BNT162b1) [62] or Moderna [63].

Liposomes can be classified in two groups: (i) multilamellar vesicles (MLV), which are spontaneously formed when a lamellar-inducing lipid mixture is dispersed in an aqueous medium; or (ii) unilamellar vesicles, which in most cases come from MLV but require further treatment, and are classified by size into small, large, or giant unilamellar vesicles. For instance, giant unilamellar vesicles (GUV, >1 micron diameter) are often used under fluorescence confocal microscopy, combined with fluorescent probes [64,65]. Large unilamellar vesicles (LUV, from 1 micron to 100 nm diameter) are useful for membrane permeation (leakage) assays [66]. Small unilamellar vesicles (SUV, <100 nm diameter), often obtained by sonication of MLV [67], present a higher lateral tension and are used to evaluate membrane fusion processes [68] and to prepare supported planar bilayers (SPB) [69,70], that will be discussed later. Liposomes can also be combined with proteins (proteoliposomes) to achieve further complexity [71].

## 4. Membrane Biophysics: Techniques

If there are still open questions in the field of bioactive lipid functionality, lipid biophysics definitely has its own share of enigmas. Perhaps the most intriguing question in this particular field regards lipid domains/platforms in vivo: When do they appear? Where exactly? How can we quantify them? These questions reflect the aforementioned problems presented by living cell membranes: if domains are transient—i.e., short-lived—we need a technique with great temporal resolution, therefore discarding powerful imaging tools such as electron microscopy, due to the requirement for sample fixation. If domains appear not in cell membranes but in organelle membranes, then we require a technique capable of making that distinction locally, as distinct from bulk measurements. Furthermore, if domain sizes are at the nanoscale, conventional microscopes will not be useful due to the resolution limit of optical microscopy. To this extent, many efforts are being made using super-resolution techniques such as STED microscopy and STED-FCS systems to measure nanoscale diffusion mechanics [72,73,74], and improvements in technology will eventually enable us to find the answers about membrane nanostructures in living cell membranes.

Fortunately, model membranes are much more stable and easier to control, which enables more techniques to be efficiently used on them. For lipid membrane characterization, the most commonly used techniques have been: (i) calorimetry, particularly differential scanning calorimetry [75] and (ii) fluorescence, either direct imaging through confocal microscopy [76], or more advanced quantitative systems such as FCS [77] or life-time spectroscopy [78], among many others. Over the last decade, molecular dynamics simulations have also gained relevance in the field [47,79,80], and, as the lipid membrane concept is extremely plastic and the bilayer can also be interpreted as a biomaterial, the field is also open for other techniques from different physics-related fields. Thus, techniques such as positron annihilation lifetime spectroscopy have been applied successfully to membrane characterization [81,82]. Another physical tool that has acquired great predominance in the field is atomic force microscopy (AFM) [83], which is a kind of multi-purpose scanning probe microscopy conceptually derived from the scanning tunnel microscopy (STM), but with great advantages for biological applications, the most important ones being the possibility of scanning aqueous samples [84] and the capacity for non-charged sample scanning. These specifications soon attracted the attention of bioscientists and, specifically, of membrane biophysicists. Moreover, AFM-based techniques have been further developed for biology-related purposes ever since, from magnetic force microscopy [85] to more recent improvements like scanning dielectric microscopy, which allows non-invasive and label-free measurements of liposome lamellarity at the single liposome level [86]. This demonstrates the impact of AFM-related techniques in the biophysics field and further improvements are expected in the near future.

**Figure 3:**
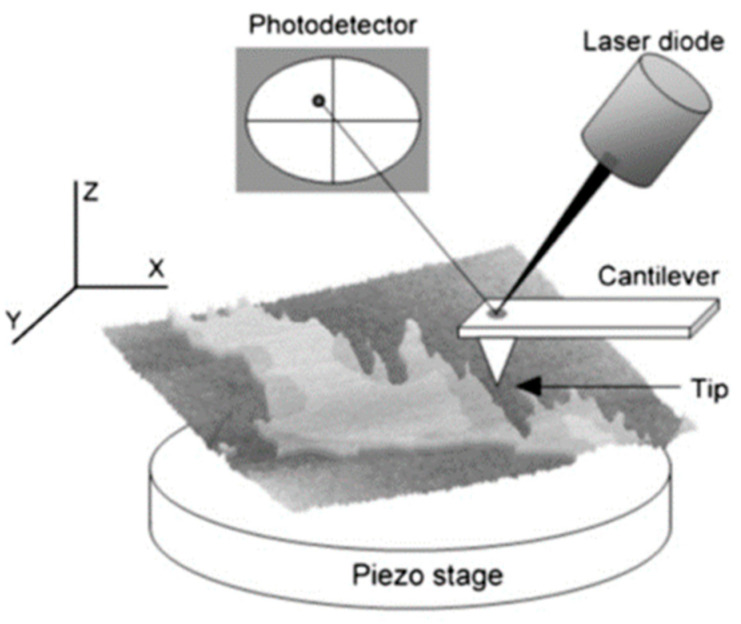
Overview of the basic parts of an AFM. A laser is focused on the tip that reflects the beam towards a photodetector. Tip–surface interactions bend the cantilever, which in turn generates a laser beam displacement. Then, the photodetector and the AFM software transform laser movements into the desired information. From Garcia-Manyes and Sanz [88].

The basic parts of an AFM setup are shown in Figure 3. In summary, the sample is scanned by a tip attached to a cantilever, which is focused by a laser beam. When a change in the height profile of the surface (Z axis) is encountered, the cantilever bends accordingly and this causes a deflection of the laser. This deflection is quantified by a photodetector and interpreted, with the supporting software included in the AFM. Depending on the mode of use, the AFM interface will generate by itself the result—e.g., an image or a force curve.

Another interesting feature of AFM usage is the availability of different modes for data retrieval, particularly during image acquisition, depending on the nature of the sample under study, and on tip–surface interactions. The ‘contact mode’ is commonly used, in it the tip is constantly in contact with the sample. However, in biological samples, this mode may be intrusive as, depending on the applied force and the nature of our sample, the tip can damage the sample and dramatically affect its integrity: objects may be displaced from their original position or partially disassembled. For this purpose, a more convenient mode would be the use of intermittent contact, such as tapping or other more advanced methods that combine imaging and force spectroscopy, which will be later described.

Due to the scanning probe nature of the AFM, it also exhibits some methodological problems or liabilities. AFM tips can spontaneously ‘pick up’ debris or aggregates affecting the original properties of the tip or the cantilever, e.g., the spring constant of the cantilever may be altered (although in many cases a re-calibration of the system is enough to circumvent the issue) or the tip may lose its intended shape. Thus, tip or cantilever fouling is a recurring problem for AFM users as it often generates artifacts and requires immediate washing of the tip/cantilever or, usually, a replacement with a clean new one. Tip functionalization (i.e., the chemical modification of the AFM tip with a specific agent) is also a common way to limit undesired interactions [87].

## 5. Membranes under AFM: The Basics

The use of AFM on membrane models has acquired a particular prominence in the last two decades [70,88]. The basic principle was to form SUV of a defined composition and then extend them to form SPBs (vesicle adsorption method), as previously mentioned. However, different methodologies were developed later, such as the direct spin-coating of lipids onto the support surface [89]. This is of interest as the vesicle adsorption method requires heating of the SUV over the T_m_, which in some samples may be difficult to achieve, particularly if the mixture presents a high concentration of saturated long chain ceramides, because of their high T_m_ values [90]. Another advantage of the alternative spin-coating method is the absence of divalent cations, which are required in the vesicle adsorption method and, if not washed properly afterwards, may affect measurements [91,92]. Sample preparation methods are in constant improvement as in most cases protocol optimization is the easiest way to increase the quality of an experiment. For instance, SPB preparation may lead (depending on the chosen method, the details of the protocol, and the nature of the lipids involved) to inefficient support coverage, as bilayers appear as distinct ‘patches’, sometimes with insufficient area to perform reproducible measurements and generating artifacts due to inefficient lipid mixing if two or more lipids are present. These small patches often exhibit, for instance, abnormal thicknesses [93], pointing to the presence of an artifact. A common way to improve bilayer extensions for the vesicle adsorption method is the use of divalent cations, typically calcium (II) [94,95,96], although in many cases these cations require to be washed away after SPB formation, as previously mentioned. SPB are also required to exhibit the lowest possible roughness in order to decrease any background ‘noise’, this being particularly important in high-resolution AFM imaging. Thus, the nature of the support is also a key factor, and SPB experiments in most cases use atomically flat mica sheets, while supports such as silicon, HOPG (highly oriented pyrolytic graphite) [97] or silicon oxide are used for AFM characterization of other types of biological samples.

**Figure 4:**
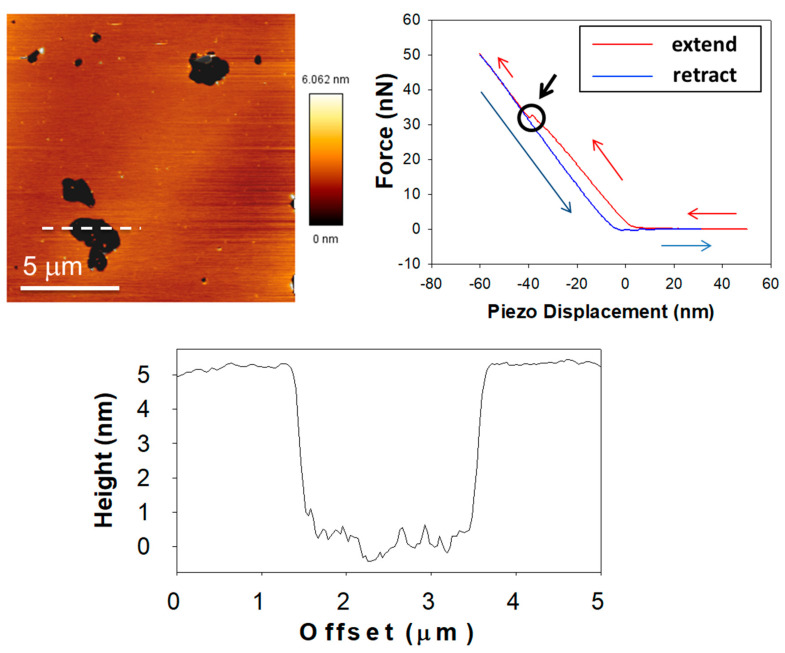
Classic experimental approach for SPB characterization under the AFM. Upper-left panel shows an AFM image of a lipid bilayer in a gel phase (pSM:Chol:pCer 54:23:23, as published in García-Arribas, et al. [30]). The height profile of the dashed line is depicted in the bottom panel; the black area is a bilayer defect that allows direct quantification of bilayer thickness (≈5 nm). The upper-right panel is a force-distance curve where the extension is colored in red and the retraction in blue. The circle indicated by the black arrow highlights the breakthrough event, the latter marking the force required to pierce through the bilayer (which is related to bilayer stiffness).

What is the precise information retrieved when AFM is applied to membranes? What is the particular purpose of this technique? AFM, as a scanning probe technique, can provide information on two general aspects: topography and nanomechanics. These two are respectively linked to image acquisition and force spectroscopy. On one hand, AFM provides direct images of any surface under scan, through different modes (contact, non-contact, intermittent contact…). On the other hand, AFM can ‘touch’ the surface to test its nanomechanical properties (nanomechanical resistance, elasticity…). These modes are not mutually exclusive, as image acquisition may be followed by localized force spectroscopy on a specific point of the previous image, but a stable sample is required. Thus, AFM provides information about membrane thickness (including the presence of segregated domain phases and the detection of proteins, characterizing subunits in some cases [98]) and about membrane stiffness (localized, for example, for every phase present [99]). Figure 4 shows an example of this combined approach. AFM can also be associated with fluorescence-related techniques, with an appropriate setup [100].

Over the last decade, improvements in AFM technology point in the same direction, namely reducing the time required for a full characterization of any sample, not only because of the fast nature of biological processes, but also to reduce the possibility of an impact of the AFM scan on the state of the sample over time, such as: (i) sample alteration by tip contact, (ii) local heating produced by laser focalization, or (iii) (if the experiment is combined with fluorescence monitoring) fluorescent probe photobleaching by the laser.

In addition to the aforementioned problems, the time scale of conventional time-resolved AFM experiments may also present issues not necessarily related to sample alteration but associated to the AFM itself. Particularly for experiments in aqueous media, which is the case for biological experiments, laser alignment has a tendency to drift over time. Although this drift may seem a small problem at first glance, if a conventional time-resolved AFM image takes, for instance, around 10 min to complete, this means a significant difference between starting vs. final laser alignment. Why is this relevant? Because laser alignment defines the force that the AFM system applies on the sample: the drift causes the starting force to change during the experiment and it has to be constantly readjusted to keep it at the desired value (commonly the lowest possible one to avoid sample damaging, as drift usually causes higher forces to be applied) [101]. This is particularly important because once the AFM scan starts, force readjustment is also subject to drifting, which means that the adjustment has to be performed as an empirical approximation at the risk of losing contact with the sample if the force is excessively decreased.

Thus, AFM technology has evolved into more complex (faster) modes that can combine both image acquisition and force spectroscopy at the same time [102]. Another useful improvement for the technique is the development of high-speed AFM, allowing the retrieval of multiple successive images per second (while the conventional AFM usually takes some min to perform a single image) in such a way that biological processes may be monitored and recorded on videos [103]. These advances successfully overcome most of the aforementioned issues related to sample alteration or AFM drifting, although systems are still subject to methodological AFM imitations (undesired tip-sample interactions, debris attachment to the tip), so there is probably still room for further improvements in this regard.

## 6. Membranes under the AFM: Findings

It would be difficult to review the whole relevant research currently performed with AFM on membranes, within reasonable length limits, thus our contribution will focus on some specific points of interest that the AFM has revealed or confirmed, beyond what had been discovered or observed using other techniques.

One interesting aspect is related to the aforementioned variability among different membrane models. As previously explained, liposomes and liposome-derived models are most commonly used in membrane biophysics, but they are not the only ones and, even when using liposomes, there are different preparation methods, each affecting the final outcome. A recent report by Monasterio et al. [104] makes an interesting comparison of three well-established models for cell membrane analysis: vesicles obtained from lipid extracts, inside-out membrane patches and membrane blebbing. At first glance, it would be easily assumable that the most ‘different’ of the three would be the pure lipid samples. Moreover, considering that both patches and lipid-extract-derived SPBs are, indeed, supported samples, while the blebs are vesicles ‘attached’ to the surface but not fully supported, the values of bleb stiffness would be expected to be much lower than in the other two preparations. However, the study proves that bleb vesicles have the highest stiffness measured by AFM force spectroscopy, and, indeed, the biggest difference in bilayer stiffness is detected between blebs and lipid extracts. This study demonstrates, with the use of AFM, that different membrane models present clearly distinct properties.

AFM characterization of SPB has also revealed unexpected behavior even in the simplest systems. The biophysical properties of sphingolipids—e.g., sphingomyelin, or ceramide—are markedly influenced by the length of their N-acyl chain [105]. A report by Jiménez-Rojo et al. [90] showed that pure C18:0 SM SPB had the capacity to achieve a stable phase separation due to an unusual complex transition (two peaks detected in DSC thermograms). The appearance of domains when monitoring a gel-to-fluid transition with AFM had been documented earlier [106], but they were typically unstable as they represented the transition itself (one of the phases ended being predominant and the other disappeared). The capacity of a single lipid to maintain a stable phase separation at a specific temperature opens new possibilities for an increased membrane heterogeneity in living cells. Another unexpected finding, this time using force spectroscopy, appeared in a report by Balleza et al. [107], revealing that bilayers made of ‘branched’ lipids (the ether-based diphytanyl and the ester-based diphytanoyl PC), both exhibited breakthroughs at two different forces despite being apparently homogenous under the AFM. The same phenomenon had also been reported by Redondo-Morata et al. [108], albeit in a binary system: DPPC/Chol at an equimolar ratio presented two different nanomechanical resistance values, despite appearing homogenous under AFM imaging. These findings point to two possibilities: (i) two different but equally favored ways (from the point of view of thermodynamics) to achieve an AFM tip breakthrough or, perhaps more probable; (ii) nanoscopic domains below the resolution capacity of AFM tips (nominal radius typically around 20 nm). We should also consider that nanoscopic domains could be more likely to appear when two or more lipid species are present, just as in the report by Redondo-Morata et al. [108], rather than in bilayers made of a single lipid such as those studied by Balleza et al. [107].

Force spectroscopy of lipid bilayers under the AFM has also revealed a particularly interesting finding related to the capacity of both lipid leaflets to interact between them in a single bilayer. Briefly, lipid membranes are often considered a single entity, and, while leaflet asymmetry has been thoroughly documented over the last decades, the general consensus was that the whole membrane was a single physico-chemical structure, regardless of the lipid asymmetry present. Then, the idea of each lipid leaflet of a bilayer presenting different biophysical properties was suggested, and finally demonstrated in a report by Alessandrini et al. [109] in 2012. This study described that AFM force spectroscopy of lipid bilayers made of a single lipid could exhibit a two-step breakthrough. This was interpreted as the tip first piercing the proximal leaflet (the one farther away from the support, and closer to the AFM tip) and then, at a higher force, the distal leaflet (in direct contact with the support, farther from the AFM tip). The same report indicated that a SPB could exhibit either a one-step or a two-step piercing process, depending on the temperature used for the vesicle adsorption method. This phenomenon was called ‘membrane uncoupling’ and seemed to occur when using lower temperatures during bilayer formation. Interestingly, the T threshold for a lipid to form coupled or uncoupled membranes was related to T_m_ in some cases; for instance, POPE bilayers were uncoupled when formed at T = 15 °C but coupled at T = 30 °C (POPE T_m_ = 25 °C), pointing to the importance of achieving a fluid phase for a coupled SPB preparation. However, other lipids, such as POPG, exhibited uncoupled bilayers well above the T_m_ in the same report, which indicates that not every fluid lipid gives rise to coupled mechanics. The same study also pointed to AFM tip velocity as a factor to detect uncoupled bilayers, as higher tip speeds showed but a single event, which the authors explained by an increased friction between leaflets.

The relevance of the unexpected membrane uncoupling was clear, as it demonstrated that different leaflets of a single bilayer could have different properties, and the support could exert a stiffening effect on the distal leaflet. It also meant that the membrane was able to achieve a metastable state when the tip was piercing the first leaflet only, demonstrating that the distal leaflet was not immediately collapsing. Membrane uncoupling was later detected in samples formed by the spin-coating procedure, and, in some cases, it was even detected for both the spin-coating and the vesicle adsorption procedures of the same lipid sample, which essentially discarded any contribution caused by the divalent cations used in the vesicle adsorption method [30]. In addition, lipid samples that could exhibit membrane uncoupling included pure gel-phase samples and also liquid-ordered ones, demonstrating that the T_m_ dependency was not as clear as previously presumed [30]. All these results point to a complex nanomechanical behavior of SPB, resulting from at least (i) the preparation procedures; (ii) the nature of the lipids; (iii) the properties of the support; and (iv) an important contribution from the instrumental setup and settings. This issue was thoroughly analyzed in a report by Relat-Goberna et al. [110]. A more recent report by Vázquez et al. [111] showed that lipid leaflets could become locally uncoupled after preparation, thus inducing bilayer asymmetry. This report points to lipid phase mismatch between leaflets as an additional factor to bilayer uncoupling, which greatly increases the complexity of these events as lipid phases may be locally segregated not only laterally as we would initially think, but also in an asymmetrical fashion along each leaflet. This ‘local uncoupling’ presents itself as a novel, interesting topic in membrane heterogeneity studies. Hopefully, further research will focus on the impact of this phenomenon on lipid–protein interactions within the membrane, particularly for transmembrane proteins.

Regarding lipid–protein interactions, a recent report from Banerjee and Lyubchenko [93] showed the importance of the presence of a SPB during the amyloid fibril (Aβ42) formation process. Aβ42 reportedly adsorbs to POPC-containing bilayers, without being inserted [112]. Using an optimized protocol for multicomponent SPB preparation in order to have smooth and large lipid extensions on the mica support, Banerjee and Lyubchenko [93] monitored on-membrane aggregation of protofibrils under the AFM. Interestingly, the aggregation process of oligomers generated the formation of protofibrils, and through quantitative analysis of AFM images, the authors demonstrated that these protofibrils exhibited a shorter length when compared to the mature solution-generated fibrils. In addition, on-membrane generated protofibrils appeared straight, non-twisted and non-branched. This is relevant because it supports the hypothesis of the governing role of membrane and protein-lipid interaction for Aβ42 fibril formation.

## 7. Impact on Membrane Biophysics ‘Hot Topics’

The influence of the support in SPB has been a hot topic of research, not only because of the nature of SPB as model systems, but also due to the great underlying question of ‘how does cytoskeleton affect living membranes?’ This is not an easy question to answer for membrane biophysicists, as most membrane models are unsupported or, if supported, are not cytoskeleton-bound. In this particular instance, the use of supported models (such as SPB) may be more interesting than unsupported ones, as they could to some extent reproduce some of the effects that cytoskeleton could exert [113]. Not unexpectedly, different supports show different properties. Reports indicate that rougher supports tend to decrease the size of segregated phases in a lipid SPB [114,115]. This is a relevant finding because it could explain why the size of lipid domains exhibits such a great variability between different models: liposomes (such as GUV) present domains of up to several microns [31], while mica-supported bilayers visualized by either AFM or fluorescence often exhibit domains up to 10 times larger [30,116].

Domain size is also a recurrent topic of discussion, as one of the main criticisms received by the ‘lipid raft’ hypothesis is the current near-impossibility of directly visualizing lipid domains on living cell membranes, while in model systems they can be clearly assessed. This has been traditionally explained by the transient nature of the domains and the nanoscopic size, below the resolution limit of many techniques. Regarding the latter, there is a further hypothesis that states the possibility that the cytoskeleton is the cause of the small size of the domains, as the cytoskeleton would define ‘boundaries’ within the membrane (‘picket fence model’ [117]) and the domains would appear at the contact points between lipids and the cytoskeleton meshwork [114]. Recent reports on membrane diffusion dynamics further support this hypothesis [72].

It should be noted that some of these phenomena have already appeared in non-cytoskeleton supported bilayers as well. As already stated, previous reports by Balleza et al. [107] and Redondo-Morata et al. [108] indicate that nanoscopic domains are a possibility. It should also be taken into consideration that some of the previously described findings, such as multi-step breakthrough events due to bilayer uncoupling [30,109,110,111] or two-modal breakthrough events in apparently homogenous samples [107,108], are almost certainly related to the fact that SPB are, indeed, supported. Would these events also happen in cytoskeleton-supported bilayers? Further experiments will probably try to answer this question in the future, but it seems a reasonable assumption that if nanodomains are present in both SPB and cytoskeleton-based bilayers, then cytoskeleton may also have a role in local membrane uncoupling [111] as the leaflet in contact with the cytoskeleton will likely have some different biophysical properties than the opposite one.

**Figure 5:**
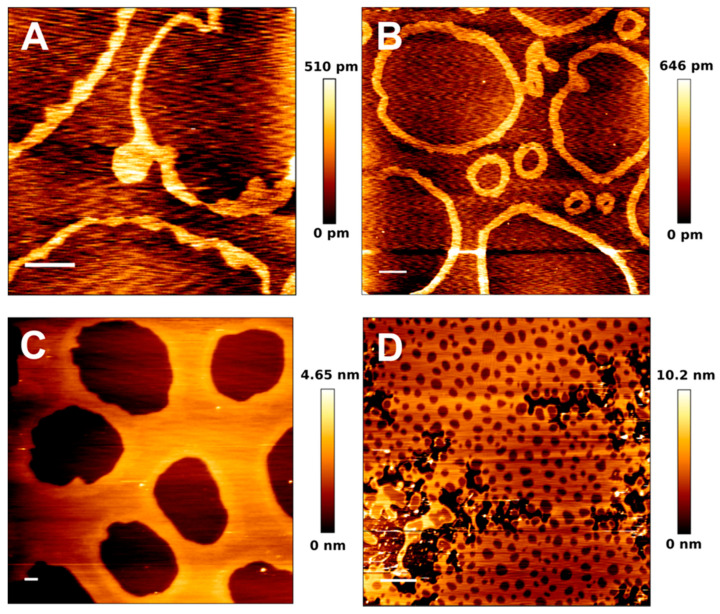
AFM images of pSM:pCer:Chol-based SPB at different mol ratios. pSM:pCer:Chol (54:23:23) (**A**), (60:20:20) (**B**), (66:17:17) (**C**), and (70:15:15) (**D**). Scale bars: 1 μm. Panels A and B show the spontaneous formation of ‘ring-like’ phases in absence of proteins. Adapted from González-Ramírez et al. [99].

Regarding non-cytoskeleton supported SPB, a recent publication from our lab [34] also demonstrated, using AFM, that domains might be unstable in SPB for some lipid mixtures (related to the combined presence of C24:0 and C24:1 sphingolipids), to the point of completely disappearing over time, while non-supported liposomes of the same composition were perfectly stable. This would point to a support-driven effect that reduces domain size over time until they become undetectable by AFM imaging (the possibility of nanodomains under the AFM resolution limit cannot be discarded). Furthermore, another AFM study revealed recently that some lipid compositions of sphingolipids and cholesterol may form ring-like phases (Figure 5), even in the absence of any cytoskeleton molecule [99]. These ring-like gel phases greatly affected the nanomechanical properties of the lipid phase ‘inside’ the boundary defined by the ring, increasing the resistance to a point that the AFM tip was unable to pierce through it, indicating an extremely packed and stiff phase [118,119]. All these results considered, the influence of supports is undeniable and the complexity of the supported lipids even in the absence of proteins cannot be underestimated.

## 8. Conclusions and Perspectives

The use of AFM in membrane biophysics, and particularly in the study of lipid self-assemblies, has helped in gaining a more thorough understanding on how membranes are built. However, more importantly perhaps, it has also pointed to new relevant details and to a higher degree of complexity in totally unexpected issues that went previously undetected due to the limitations of other techniques. Moreover, as AFM technology evolves towards faster and better resolved systems, supported by advanced imaging techniques such as STED, we should expect that these issues are solved and suitable answers are conveniently found for our questions. The increase in complexity of the samples under study, as well as the improvements in the scanning techniques, will probably generate new, unexpected questions, as further research targets will surely appear.
